# Anesthesia for the First Fully Robotic Simultaneous Living Donor Liver Transplant in Europe

**DOI:** 10.7759/cureus.101604

**Published:** 2026-01-15

**Authors:** Filipe André Pereira, Filipe Pissarra, Rita Poeira, Paula Rocha, Margarida Canas, Sandra Dias, Susana Cadilha, Hugo Pinto Marques

**Affiliations:** 1 Department of Anaesthesiology, Unidade Local de Saúde de S. José - Hospital Curry Cabral, Lisbon, PRT; 2 Department of General Surgery, Unidade Local de Saúde de S. José - Hospital Curry Cabral, Lisbon, PRT

**Keywords:** anesthesia, ischemia time, liver transplantation, living donor, robotic surgery

## Abstract

Robotic surgery is progressively redefining the scope of minimally invasive procedures, yet its role in liver transplantation remains limited. While robotic techniques have been successfully applied to living donor hepatectomy and, more recently, to deceased donor transplantation, fully robotic simultaneous living donor liver transplantation (LDLT) has not previously been reported in Europe. We present the anesthetic management of the first such case, performed in parallel operating rooms with dual robotic platforms. A 64-year-old female with hepatitis C virus-related cirrhosis and hepatocellular carcinoma underwent robotic orthotopic liver transplantation with a right lobe graft from her 38-year-old daughter. Both procedures were completed without conversion to open surgery. Careful anesthetic planning, meticulous hemodynamic control, and cross-team coordination allowed minimization of ischemia times and excellent early postoperative outcomes for both donor and recipient. This case underscores the feasibility and safety of simultaneous fully robotic LDLT and highlights the unique anesthetic challenges inherent to this novel approach.

## Introduction

Robotic technology has progressively reshaped minimally invasive surgery by providing superior dexterity, enhanced instrument articulation, tremor filtration, and improved ergonomics, which facilitate complex procedures in anatomically challenging regions such as the hepatobiliary tract compared to conventional laparoscopy [1].

Large studies show that robotic liver and pancreatic resections result in lower intraoperative blood loss and shorter length of stay, with comparable or reduced complication rates and conversion-to-open rates when compared to conventional laparoscopy [2-5]. Specifically, robotic liver resections have been associated with less blood loss (median 30 vs. 100 mL) and shorter hospital stays (median 3 vs. 4 days) compared to laparoscopic approaches [3].

In liver transplantation, robotic applications initially centered on living donor hepatectomy, while recipient hepatectomy and graft implantation remained open procedures. The first fully robotic orthotopic liver transplantation in a human was reported in Asia in 2021 [6], followed by North America in 2023 [7], both involving deceased donors. More recently, in 2024, the first European series of full robotic whole-graft liver transplantation with deceased donors was published. The study established patient selection criteria for robotic liver transplantation, including hepatocellular carcinoma in liver cirrhosis with a small caudate lobe (posterior hepatic segment I), low degree of portal hypertension, absence of porto-mesenteric thrombosis, and low Model for End-Stage Liver Disease scores [8,9].

The extension of robotic techniques to both donor and recipient operations in living donor liver transplantation (LDLT) represents the next frontier in transplantation surgery. We report the first case in Europe of a simultaneous fully robotic LDLT performed in parallel operating rooms. This milestone brings about implications not only for surgical technique, including reduced incision size, improved postoperative recovery, enhanced surgical precision, and potentially reduced ischemia time, but also for anesthetic management, given the unprecedented complexity of coordinating two concurrent high-acuity operations [5,8,9]. The simultaneous robotic approach introduces unique logistical and clinical challenges, including the need for dual anesthesia teams with expertise in both robotic surgery considerations and transplant physiology, coordination of pneumoperitoneum management in both donor and recipient, timing of graft extraction and implantation to minimize ischemia, and management of hemodynamic changes associated with prolonged pneumoperitoneum and steep positioning in both patients [8,9]. This case demonstrates the feasibility of applying robotic technology to the most complex aspects of LDLT while potentially maximizing the benefits of minimally invasive surgery for both donor and recipient.

## Case presentation

A 64-year-old woman with hepatitis C-related cirrhosis and hepatocellular carcinoma was listed for LDLT. Her only comorbidity was well-controlled hypertension. Her 38-year-old daughter, with an unremarkable medical history and normal laboratory results, was accepted as a donor after a thorough multidisciplinary evaluation. Both patients provided informed consent for a fully robotic simultaneous approach. The procedures were conducted in two adjacent operating rooms, each equipped with a dedicated robotic surgical system (Da Vinci system). Two surgical and anesthesiology teams were assigned to the donor and recipient, enabling coordinated monitoring of progress in both operating rooms.

Donor procedure

Under general balanced anesthesia with sevoflurane, the donor underwent a fully robotic right hepatectomy. A single-lumen midline catheter was placed in the left basilic vein, with two peripheral venous catheters (16G and 20G). In addition to standard ASA monitoring, a left-sided radial arterial line connected to the ProAQT system, bispectral index monitoring, and neuromuscular blockade monitoring were used. Pneumoperitoneum was maintained at 12 mmHg, with a 15º reverse Trendelenburg position. Indocyanine green fluorescence delineated segmental anatomy. 3000 mL of balanced crystalloid solution was infused with an estimated blood loss of around 100 mL, with no need for blood transfusion or vasopressor use throughout. The graft was extracted through a Pfannenstiel incision. Multimodal analgesia consisted of fentanyl (0.2 mg), ketamine (30 mg), paracetamol (1 g), dexmedetomidine (60 µg; 1 µg/kg), metamizole (2 g), parecoxib (40 mg), tramadol (100 mg), and a laparoscopic-assisted bilateral four-quadrant transversus abdominis plane block using 10 mL of 0.375% ropivacaine per quadrant [10]. Hepatic clamping and explantation were deferred until the recipient was fully prepared in order to minimize ischemia time, leading to a total donor operative time of 495 minutes. Prophylaxis for postoperative nausea and vomiting consisted of dexamethasone (4 mg), ondansetron (4 mg), and droperidol (0.625 mg). Neuromuscular block was reversed using sugammadex until a TOF ratio of 0.9 was achieved. The donor was extubated in the operating room and transferred to the intensive care unit, where she remained for observation for 24 hours.

Recipient procedure

Simultaneously, the recipient underwent total robotic recipient hepatectomy using Belghiti’s modified piggyback technique under general anesthesia with sevoflurane. A 5-lumen right internal jugular central venous catheter and two peripheral venous catheters (14G and 18G) were placed, as well as a radial and femoral arterial line, connected to the PiCCO2 system. Additionally, bispectral index and neuromuscular block monitoring were used throughout. Pneumoperitoneum was maintained at 12 mmHg with a 15º reverse Trendelenburg position. 5000 mL of balanced crystalloid solution was infused with an estimated blood loss of 150 mL with no need for blood transfusion. A transient noradrenaline infusion was required during the peri-unclamping period, with a maximum dose of 0.2 µg/kg/min. The liver was extracted, and the graft was subsequently introduced through the camera port incision, vertically enlarged to 10 centimeters. Implantation of the liver graft then began, starting with side-to-side anastomosis of the inferior vena cava, followed by arterial and biliary anastomosis. Cold ischemia time was 15 minutes, with a warm ischemia time of 56 minutes. Intraoperative hepatic artery thrombosis occurred, requiring thrombectomy and a redo-anastomosis. Total operative time was 540 minutes. The patient was extubated at the end of the surgery and transferred to the intensive care unit. Intraoperative immunosuppression consisted of methylprednisolone (180 mg) administered at induction and repeated after vascular unclamping, together with basiliximab (20 mg) given after unclamping. Multimodal analgesia included a continuous fentanyl infusion (0.8 µg/kg/h) throughout the procedure, metamizole (2 g), paracetamol (1 g), and morphine (5 mg). Prophylaxis for postoperative nausea and vomiting consisted of ondansetron (4 mg). Neuromuscular block was reversed using sugammadex until a TOF ratio of 0.9 was achieved.

Postoperative course

The donor’s postoperative course was uneventful, and the donor was discharged home on postoperative day three.

The recipient began heparin infusion titrated to an APTT ratio due to the intraoperative artery thrombosis and remained in the intensive care unit for three days for observation, not requiring any interventive organ support. It was then switched to enoxaparin twice daily, and she was transferred to the transplant unit, being discharged on postoperative day nine (Table 1).

**Table 1:** Anesthetic management: donor vs. recipient ASA: American Society of Anesthesiologists, BIS: Bispectral Index, G: Gauge, ICU: Intensive Care Unit, mL: Milliliters, POD: Postoperative Day, PiCCO: Pulse index Continuous Cardiac Output, ProAQT: Pulse contour cardiac output monitoring system, TAP block: Transversus Abdominis Plane block, µg/kg/min: Micrograms per kilogram per minute

		Donor	Recipient
Monitoring besides ASA standard		ProAQT, BIS, neuromuscular block	PiCCO, BIS, neuromuscular block
Vascular access	Peripheral venous access	16G + 20G	14G + 18G
	Central venous access	Single-lumen midline basilic vein acess	5 lumen central venous catheter
	Arterial access	Radial arterial line	Radial and femoral arterial line
Fluid therapy		3000 mL crystalloid	5000 mL crystalloid
Blood loss		100 mL	150mL
Blood products needed		None	None
Vasopressors use		None	Noradrenaline max 0.2 µg/kg/min
Analgesia		Fentanyl bolus, Ketamine, Paracetamol, Dexmedetomidine, Metamizole, Parecoxib, Tramadol, Laparoscopic-assisted bilateral four-quadrant TAP block	Fentanyl infusion, Metamizole, Paracetamol, Morphine
Ischemia time cold/warm		None	Cold 15 min/ Warm 56 min
Extubation at end of procedure		Yes	Yes
Total operative time		495 min (deferred hepatic clamping)	540 min
Post operative outcome		ICU 24 hours, discharge POD 3	ICU 3 days, discharge POD 9

## Discussion

This case report describes the anesthetic management of the first simultaneous fully robotic living donor liver transplant. Beyond the surgical milestone, the primary anesthetic contribution of this case lies in the coordinated management of two simultaneous major procedures aimed at minimizing graft cold ischemia time while preserving donor and recipient safety [11,12]. By providing a detailed description of the anesthetic management of both the donor and recipient, this report may help standardize perioperative care and thereby facilitate broader adoption and more frequent performance of this procedure.

Robotic surgery is revolutionizing surgical interventions, offering a minimally invasive approach to traditional open surgeries [9]. The benefits have been highlighted in several studies, including less postoperative pain, fewer postoperative wound complications, less blood loss, and shorter hospital stays [5,13,14]. The first four fully robotic liver transplants performed at this institution were described in a previous publication [8]; to date, a total of 18 such transplants have been performed.

From an anesthesiologist’s perspective, simultaneous fully robotic LDLT presents distinct challenges. Two complex procedures are performed concurrently, necessitating meticulous coordination between surgical and anesthesiology teams. The anesthetic considerations extend beyond the technical demands of each individual operation to include strategic planning for donor safety, graft preservation, minimization of ischemia time, and maintenance of optimal hemodynamic stability in the recipient during reperfusion [15]. The requirement to maintain low central venous pressure in the donor to minimize blood loss contrasts with the need to optimize preload in the recipient prior to reperfusion in a hemodynamically fragile cirrhotic recipient [15,16]. 

The parallel fully robotic approach conferred a key advantage: unprecedented minimization of cold ischemia time (15 minutes). This represents a dramatic improvement compared to deceased donor liver transplantation (average >300 minutes in our center) or sequential LDLT. In contrast, a previous report of a hybrid minimally invasive recipient procedure (laparoscopic explant followed by robot-assisted engraftment) described much longer ischemia intervals, with a warm ischemia time of 87 minutes and a cold ischemia time of 220 minutes, and substantial estimated blood loss (3600 mL) [17]. In the first series of fully robotic recipient living donor liver transplants, the median total operative time was 10 hours (robotic donor and robotic recipient combined) with a median hospital stay of 13 days [6]. In large multi-approach prospective registry data, robotic donor hepatectomy was associated with the lowest intraoperative blood loss (median 70 mL, interquartile range 50-100 mL) compared with laparoscopic and open approaches, shorter postoperative recovery, and very low complication rates [4]. From the anesthetic standpoint, minimizing graft ischemia time attenuates the metabolic and inflammatory burden associated with reperfusion, thereby reducing the risk of ischemia-reperfusion injury and supporting improved early graft function [18,19].

However, this innovation also introduces unique challenges. It necessitates the availability of two robotic platforms, highly experienced surgeons and anesthesiologists, and institutional resources generally limited to high-volume centers. Furthermore, the constraints of robotic surgery limit immediate manual intervention, thereby heightening the importance of anesthetic vigilance and comprehensive perioperative preparedness.

Beyond the technical considerations, this case suggests that simultaneous robotic LDLT may signal a paradigm shift in transplantation. If validated in larger series, this approach could improve graft outcomes, accelerate recovery, and redefine the coordination between donor and recipient procedures. Ultimately, from an anesthetic perspective, this approach demands new paradigms of communication, monitoring, and resource allocation to ensure safe and reproducible implementation.

## Conclusions

Simultaneous fully robotic LDLT is feasible, safe, and associated with excellent early outcomes in both donor and recipient when performed in a high-volume center with experienced multidisciplinary teams. This approach not only expands the scope of robotic transplantation but also introduces a new anesthetic frontier, balancing competing hemodynamic requirements, synchronizing two complex procedures, and exploiting the potential to reduce ischemia times.

While longer-term outcomes and cost-effectiveness remain to be established, this case illustrates how robotics may reshape the practice of living donor liver transplantation. For anesthesiologists, it highlights the need for proactive planning, close interdisciplinary communication, and development of specific expertise in managing robotic transplant physiology. As access to robotic platforms expands, simultaneous LDLT may evolve from an innovative milestone into a reproducible practice in selected centers, suggesting a potential paradigm shift, pending validation in larger series.”
